# Public involvement in big data projects: an ethnographically-informed study.

**DOI:** 10.23889/ijpds.v7i3.1993

**Published:** 2022-08-25

**Authors:** Kirsteen Campbell, Rebecca Whitehorn, Andy Boyd, Robin Flaig, Stela McLachlan, Christie Levein, Jacqueline Oakley, Sarah Chave, Michael Gregg, Della Ogunleye

**Affiliations:** 1 UK Longitudinal Linkage Collaboration, University of Edinburgh; 2 UK Longitudinal Linkage Collaboration, University of Bristol; 3 UK Longitudinal Linkage Collaboration, Public Advisory Group

## Objectives

UK longitudinal population studies have complex governance structures and participant safeguards. As a new Trusted Research Environment (TRE) for >20 longitudinal studies, the UK Longitudinal Linkage Collaboration (UK LLC) must ensure effective public/participant input to make central design and operational decisions whilst retaining participant trust and acceptability.

## Approach

The UK LLC’s governance model is based on the principle that participant trust lies with their study; not supporting infrastructure providers. Therefore, the UK LLC takes responsibility for system governance and data sharing agreements whilst studies retain control over participant data and its uses. Our public/participant model reflects this. We have established centralised groups to advise on data access applications and on UK LLC communications. Simultaneously we work with study managers to gain insight from their involvement work and also commission UK LLC specific activities. Including participants as well as the public helps elicit a range of input and views.

## Results

DWe have integrated public/participant input across the UK LLC by recruiting: a public panel to review applications; an advisory panel to contribute to our methodological development; and public/participant advisors to sit on the UK LLC Strategic Advisory Committee. We also work with studies’ involvement managers to draw insights from, and coordinate new, study-level involvement.

This has informed the development of data application frameworks and brings a public viewpoint to decisions regarding linkage of functionally anonymous participant NHS records for research. The involvement of study participant groups has shaped the design of data flows into the TRE (e.g., where some studies do not permit the flow of address data for geospatial research) and has helped set a ‘social contract’ regarding acceptable UK LLC data use.

## Conclusion

Working with public/participant representatives directly, and in partnership with study managers, we have developed safeguards and integrated a public/participant voice into key UK LLC activities. Our ongoing aim is to develop a sustainable new-way-of-working for record linkage in longitudinal research which is acceptable to participants and the public.

